# The Representative Meeting of the British Medical Association

**Published:** 1916-08-12

**Authors:** 


### THE REPRESENTATIVE MEETING OF THE BRITISH MEDICAL ASSOCIATION.

Judged from the gallery, the recent meeting of the Representative Body of the British Medical Association merits some substantial compliments. The proceedings were characterised by business-like method and were closely directed to practical ends. The Chairman showed that he knew the lengthy and complicated agenda thoroughly, and he ruled his audience effectively but with a light and happy hand. Generally speaking, the debates were well directed and the speeches were brief and to the point. There were some sharp differences of opinion, but the unpopular minority had its opportunity and was treated with patience and respect. Altogether the meeting was a success and reflected credit on the practical judgment of the profession.

### The New President.

Perhaps the most enthusiastic moment was the reception of Sir Clifford Allbutt, the President, whose vigorous carriage and musical voice seemed to contradict his eighty years and long record of service. A very warm welcome was given also to the Chairman of the Central War Committee and to the officials who have been responsible for the extra duties imposed by trying times. The Honorary Treasurer, Dr. Edwin Rayner, who retires after many years of service, received a special vote of thanks; and it is due to him to recognise that he leaves the finances of the Association in a satisfactory condition in spite of the special charges incurred in connection with the opposition to the Insurance Act and with the organisation of the profession relative to the needs of the Army. He is to be succeeded in his office by Dr. Haslip, who has had a long practical experience of the various departments of the Association. In connection with the finances an energetic endeavour was made to resolve that the expenses of the War Committee should be claimed from the Government. In reply, it was urged that the Committee was in a much more independent position, and was more free for criticism and remonstrance as a result of the absence of any official subsidy, and the meeting by a large majority insisted on the patriotic course of meeting the whole of the expenses from the Association's exchequer. An argument that such charges were *ultra vires* received no countenance from the solicitor, and the representatives warmly supported this conclusion. The one unfortunate feature of the record is that the Association has still to deplore a declining membership. Evidently the results of the Insurance controversy have not yet exhausted themselves, and discontented individuals show their dissatisfaction by severing their connection with the Association. Its critics are both numerous and vocal, but they do not succeed in organising any effective opposition, and possibly they might effect more by argument directed from within rather than by condemnation proclaimed from the outside. The Association shows some failure in its inability to win them, and in this respect might develop a little more tact. Discontent has usually some measure of justification.

### State Medical Service.

One of the most marked features of the recent meeting was the display of a strong disposition to resent the interference of the State with private medical practice. There was a current in the opposite direction, but it was of relatively small proportions and it manifested some inclination not to display itself too openly. The tendency here described found several opportunities of expressing itself. Certain words in the official report and in some of the official motions which seemed to imply a readiness on the part of the profession to welcome a further extension of notification to public officials of facts learned from private patients excited obvious hostility, and in some instances had to be withdrawn. The notorious suggestion that measles might be notified at a less fee than other infectious diseases did not find a single defender, and while the meeting passed no condemnation on the persons responsible for this proposal it obviously abstained more out of regard for individuals than from agreement with the method. On this point quite a number of motions involving a vote of censure had been put on the agenda paper by various divisions, but none of them were energetically pressed, and though everyone protested against the reduction of the fee as now approved by Parliament no one was able to suggest any practical method by which the position could be redeemed. The law of the land has to be obeyed by doctors as it has to be obeyed by humbler folk, and if the profession want a change in the law they must either convince or frighten the politicians. Probably the latter is the more efficacious force, but with a limited and scattered voting power medicine has not much capacity in this direction. While disposed to resist interference from the outside the representatives showed at the same time a resolution not to be led beyond their proper frontiers. Thus a motion obviously inspired by the total abstinence party and proclaiming the advisability of national teetotalism during the war met with short shrift, at least to this extent, that the meeting promptly passed to the next business. The feeling seemed to be that the matter was one for individual choice and not for collective resolution. A similar fate assailed the suggestion that, prior to marriage, everyone should be compelled to affirm the absence of "any infectious or contagious disease," and that such condition should be essential to the validity of the contract. Here, again, it seemed to be the view that the question was not one for the medical profession to decide and that interference outside the area of professional responsibilities was a mistaken policy.

